# Interventions to Improve the Reproductive Health of Undocumented Female Migrants and Refugees in Protracted Situations: A Systematic Review

**DOI:** 10.9745/GHSP-D-21-00418

**Published:** 2022-12-21

**Authors:** Silvana Larrea-Schiavon, Lucía M. Vázquez-Quesada, Lindsay R. Bartlett, Nayeli Lam-Cervantes, Pooja Sripad, Isabel Vieitez, Liliana Coutiño-Escamilla

**Affiliations:** a Population Council, Mexico City, Mexico.; b Population Council, Washington, DC, USA.; c National Institute of Public Health, Mexico City, Mexico.

## Abstract

**Objective:**

Limited evidence exists on interventions aimed at enabling reproductive health (RH) services access for undocumented female migrants and refugee women. We aimed to identify intervention characteristics and impacts on RH outcomes among migrants and refugee women in protracted situations.

**Methods:**

We conducted a systematic literature review of RH intervention studies that reported on migrants and refugee women in protracted situations. We applied 2 search strategies across 6 databases to identify peer-reviewed articles in English, Spanish, and Portuguese. Eligible studies were assessed for content and quality.

**Results:**

Of the 21,453 screened studies, we included 10 (all observational) for final data extraction. Interventions implemented among migrant and refugee women included financial support (n=2), health service delivery structure strengthening (n=4), and educational interventions (n=4). Financial support intervention studies showed that enabling women to obtain RH services for free or at a low cost promoted utilization (e.g., increased use of contraception). Interventions that established or strengthened health service delivery structures and linkage demonstrated increased prenatal visits, decreased maternal mortality, and facilitated access to other comprehensive reproductive health services, such as abortion information and counseling and postabortion care. Educational interventions indicated positive effects on RH knowledge and the importance of involving peers and meeting the unique needs of a mobile population. All intervention studies emphasized the need to accommodate migrant security concerns and cultural and linguistic needs.

**Conclusion:**

Interventions in protracted situations reported positive outcomes when they were migrant or refugee-centered and complementary, culturally acceptable, geographically proximate, and cost-sensitive, as well as recognized the concerns around legality and involved opportunities for peer learning. Free or low-cost RH services and greater availability of basic and emergency maternal and neonatal care showed the most promise but required further community outreach, education, and stronger referral mechanisms. We recommend further participatory implementation research linked to policy and programming.

## Abstract (precis)

Evidence from a systematic review shows that subsidizing health care, strengthening health services, and implementing educational interventions have positive effects on undocumented migrant women and female refugees’ sexual and reproductive health outcomes.

## Teaser

**Key Findings:**

- Providing free or low-cost services to migrant women and female refugees in protracted situations is a critical strategy in successfully ensuring access to the continuum of sexual and reproductive, maternal, and newborn care services.
- Staffing health care units with culturally competent and trained personnel influences the prevalence of contraception use and can reduce maternal and neonatal complications and adverse outcomes.
- Educational interventions can potentially have a greater impact if implemented after or concurrently with the improvement of health system capacities.

**Key Implications:**

- Implementing organizations should consider the importance of cultural sensitivity and security when designing and implementing the intervention and should support, as much as possible, a health system culture of de-identified electronic record keeping—particularly in conflict areas, as records can get lost during violent attacks.
- Implementing organizations, research partners, and target communities should be involved from the start in the design of the intervention and evaluation so that both components are responsive to community needs and have strong monitoring and evaluation methodologies to report on best practices and lessons learned.

## INTRODUCTION

International migration is a global and growing phenomenon. Its implications for health systems include challenges to ensuring necessary health and social services in transit and destination countries. It is estimated that in 2020, there were a total of 281 million international migrants worldwide (3.6% of the total population).^1^ From 2000 to 2020, the number of refugees has almost doubled, from 14 million to 26.4 million.^1^ In 2020, 48.3% of international migrants were women and girls.^1^

Undocumented migrants and refugees in protracted situations find themselves transiting locations without meeting the requirements that a sovereign state has to allow them to reside, work, and access services in its territory. Women of these populations are often isolated, without documents or support networks, and subject to gender-based violence, which is often perpetrated by their partners and family members, migration authorities, and smugglers. Living in a country that is not their own, female migrants and refugees encounter legal frameworks, discrimination, and language barriers that make it difficult for them to access basic needs such as education, health services, or humanitarian resources.^2^^,^^3^

In most countries, women who lack a regular migratory status have limited access to health services; if they are on the move, the continuum of care provision can be further restricted because of their short stay in a single geographic setting. This population is often characterized by low socioeconomic opportunity, generalized insecurity, and precarious legal status. Barriers to health care access are accompanied by unequal reproductive health (RH) outcomes among undocumented migrant women and girls and refugees in protracted situations. Once pregnant, undocumented migrant women and female refugees are exposed to inadequate antenatal care and experience a higher probability of complications during pregnancy (gestational diabetes and pregnancy hypertensive disease) and stillbirths than nonmigrant women.^4^^–^^6^ Undocumented migrant women and female refugees are also at increased risk of unintended or forced pregnancies, rape, sexually transmitted infections, and unsafe abortions.^7^ Some of the factors identified that negatively affect access to RH for this population include fear of abuse and discrimination by health professionals, limited mobility, lack of transportation and distance to the health services, and lack of adequately trained and sensitized health providers.^8^ Therefore, the intersections between migration, women, and health requires special attention to comprehensive RH service delivery, with a focus on short- and long-term contraceptive methods, sexual violence support services, abortion information and counseling and postabortion care, and antenatal and childbirth care.

Although there are interventions and guidelines aimed at targeting RH in humanitarian settings, to our knowledge, there is limited empirical evidence of the impact of these interventions on RH outcomes.^9^^–^^12^ How many people benefit and how they are cared for is regularly documented, but the results obtained are hardly systematized and subject to verification. Moreover, RH interventions are even less frequently published in peer-reviewed journals.

To address this knowledge gap, we conducted a global systematic review to identify the outcomes of intervention studies focused on improving the RH of undocumented female migrants and refugees in protracted situations. We synthesized research that highlights RH outcomes and intervention components that have been tested among undocumented migrant women and refugees in protracted situations, regardless of whether the intervention was described as successful or unsuccessful, to provide evidence and recommendations for governmental and nongovernmental organizations (NGOs) to support their goals of improving the well-being of these populations.

This study contributes to understanding how to address the RH service needs of undocumented female migrants and/or refugees in protracted situations by exploring interventions and unveiling complexities at the intersections between migration and health. These intersections are of particular interest to global and national stakeholders committed to upholding international human rights frameworks and achieving the Sustainable Development Goals, which aim to end poverty, protect the planet, and ensure prosperity and well-being for all.

This study contributes to understanding how to address the RH service needs of undocumented female migrants and/or refugees in protracted situations by exploring interventions and unveiling complexities at the intersections between migration and health.

## METHODS

We conducted a systematic literature review of intervention studies on RH following the Preferred Reporting Items for Systematic Reviews and Meta-Analysis (PRISMA) Statement guidelines. Because we aimed to identify the characteristics and impact of interventions, we included both qualitative and quantitative research in our analysis. Table 1 details the study’s inclusion and exclusion criteria.

**TABLE 1.** Inclusion and Exclusion Criteria for the Selection Process on Interventions to Improve the Reproductive Health of Undocumented Female Migrants and Refugees in Protracted Situations

Category	Included	Excluded
Population of interest	Undocumented female migrants and female refugees in a protracted situation, as defined by the International Organization for Migration (2019)	Internally displaced populations, documented migrants and/or refugees already resettled in a host country
Intervention	Any reproductive health-related intervention seeking to improve contraceptive care, safe abortion, prenatal care, childbirth care and/or postnatal care	
Control	Presence of at least 1 group likely to access the same reproductive health services were considered control or comparison	
Outcomes of interest	Primary outcomes on the reduction of barriers to care for reproductive health services (improvement in the availability, accessibility, and acceptability of reproductive health services received)	Studies that do not quantify or qualify health outcomes
	Primary maternal health outcomes reported as prevalence, incidence, rates, odds, and any other estimator that indicates the improvement (or lack) of health conditions of pregnant women	
	Primary outcome on short-term and long-term contraceptive use, reduction of unintended pregnancies, access to safe abortion services, reduction in unmet need for family planning among women of reproductive age, coverage of contraceptive services and contraceptive prevalence	Studies aimed solely at describing HIV and other sexually transmitted infections
Study design	Study designs included should: (1) have a time frame of implementation; (2) have clear outcome indicators; (3) mention a change in one or more of the reproductive health outcomes of interest for this study; and (4) be reported in qualitative, observational, or impact evaluation studies.	Studies that do not separately analyze data from undocumented female migrants or female refugees
	Study designs included were randomized control trials, nonrandomized controlled trials, controlled before-after studies, post-intervention studies, and qualitative studies	Studies that do not report results separately between undocumented female and male migrants and refugees
Publication date	January 1, 2000–December 31, 2019	
Language	English, Spanish, and Portuguese	

Following the 2019 Glossary on Migration of the International Organization for Migration, we defined an undocumented female migrant as a female nonnational who enters or stays in a country without the appropriate documentation.

We defined a female refugee using the 1951 Refugee Convention definition^13^:

*a person who, owing to a well-founded fear of persecution for reasons of race, religion, nationality, membership of a particular social group or political opinions, is outside the country of her nationality and is unable or, owing to such fear, is unwilling to avail herself of the protection of that country.*

We defined a protracted situation as a situation in which refugees have been unable to return to their habitual residence for a long period of time and where the process for finding durable solutions, such as repatriation, integration in host countries, settlement in third locations, or other mobility opportunities has stalled.^13^

We decided to include and focus on these populations, given their limited access to health care because of a lack of documentation and living and housing conditions (e.g., refugee camps, informal settlements, or migrant shelters). There is a lack of consensus on when the refugee situation is considered prolonged. Some organizations and stakeholders emphasize the duration of displacement (e.g., 12 months or 3 years) as a reference point. Others consider that the situation is prolonged if people are not able to return to their country of origin, so their location is used as the main criterion. Finally, others identify a continued need for humanitarian action as the key element of the definition. For the present study, we defined refugees in protracted situations encompassing all situations in which the process of finding solutions has stalled.^13^

We defined RH as^14^:

*a state of complete physical, mental and social-well-being and not merely the absence of disease or infirmity, in all matters relating to the reproductive system and to its functions and processes.*

To focus the review’s scope on answerable research questions related to RH interventions, we refrained from broadening our search to gender-based and sexual violence services. Because of the high prevalence and long-term consequences of gender-based and sexual violence among undocumented female migrants and refugees in protracted situations, we believe that separate systematic reviews on these issues are needed. We excluded studies that solely focused on sexually transmitted infections and not broader RH programming. Interventions were defined as an articulated or sequential set of political, programmatic, and ethical actions to improve the beneficiaries’ knowledge, skills, conditions (including health), and other competencies. These actions may be implemented by health and social service professionals or researchers and must have had the purpose of reducing a social problem, for example, lack of access to RH services.^15^ Interventions included were limited to services intending to promote the well-being of women’s reproductive lives across the continuum of care, including contraception, prenatal, childbirth, and postnatal care, and other comprehensive RH services, including abortion information and counseling and postabortion care.

### Search Strategy

**FIGURE 1:**
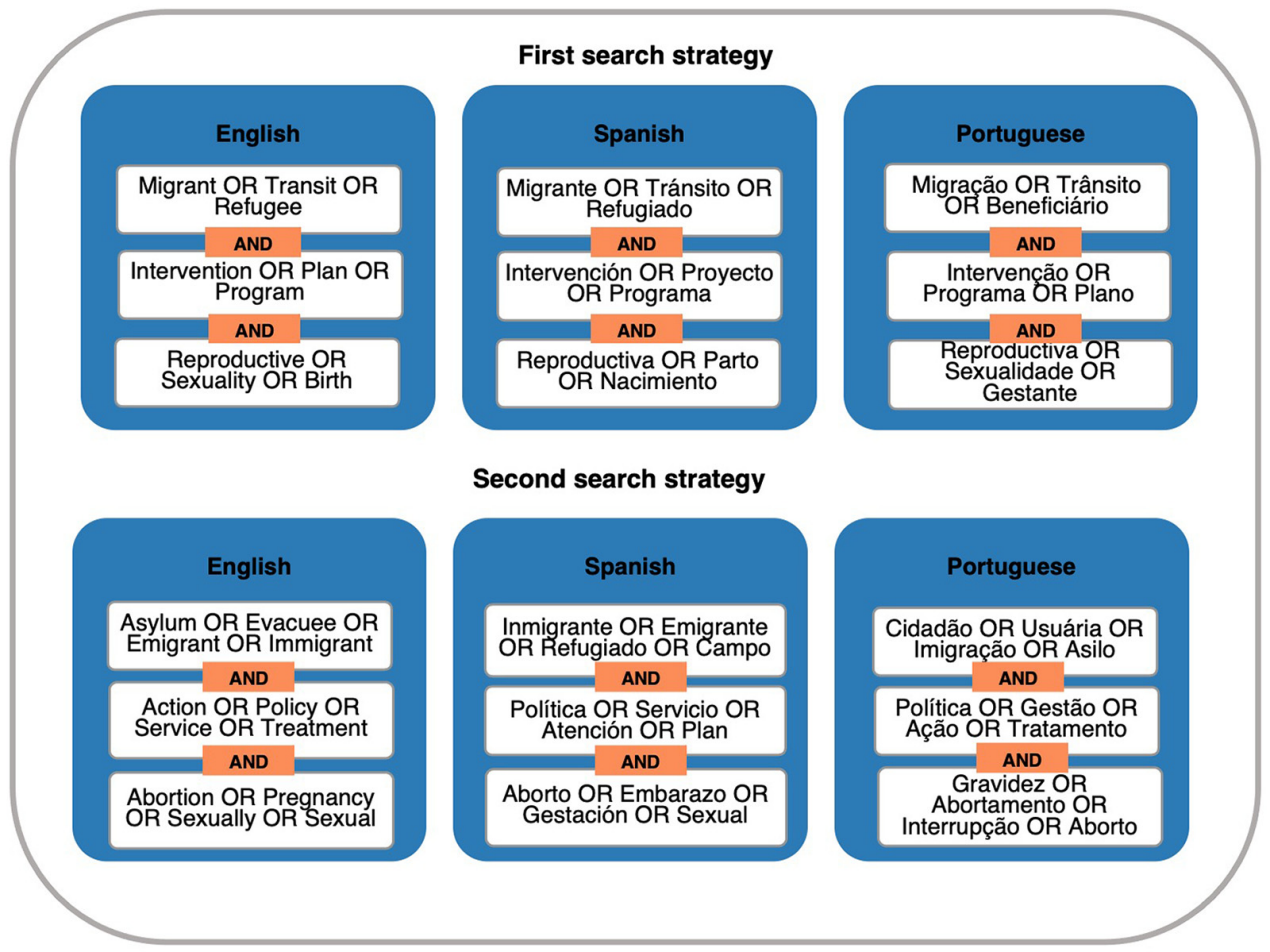
Search Strategies in English, Spanish, and Portuguese Used in the Identification of Records for the Systematic Review

We used 6 search engines specialized in health and social sciences: Cochrane Library, MedLine (via PubMed), BIREME, PsycNet, SCIELO, and Web of Science. Screening the reference list of relevant systematic reviews as well as an intentional search in Google complemented the search strategy. Boolean operators were used for those search engines that allowed it, as well as the corresponding filters by type of study, date of publication, and language (English, Spanish, and Portuguese). We used 2 search strategies for every search engine (Figure 1). Each search strategy included a combination of terms related to population, intervention, and outcome. Keywords with high sensitivity and low specificity, such as women, contraception, or contraceptive, were removed after several search iterations to identify relevant terms in each search strategy. We added the term asylum as part of our search strategy to capture some of the articles about undocumented migrants and refugees in protracted situations. However, we did not include any article that solely focused on asylum seekers. Each word combination was conducted with the corresponding translation in each language. The searches were conducted independently by NL, LB, and LC.

In Google, we added the names of NGOs, academic institutions, and United Nations (UN) agencies known to work with the populations of interest to the previously selected search terms. The NGOs and UN agencies included in the search were the UN Refugee Agency, Médicins Sans Frontieres, Médicins du Monde, EMERGENCY, Marie Stopes International, Women’s Refugee Commission, Inter-Agency Working Group on Reproductive Health in Crises, National Institutes of Health, and the Population Council. These organizations, as identified by the Population Council, have a history of providing health care services to migrant and refugee populations in humanitarian settings, documenting their interventions, and conducting monitoring and evaluation reports of their efforts.

### Data Analysis

The research team used Microsoft Excel to download all returned citations from the searches and summarize data from the studies included in the analysis. All authors reviewed data screening and analysis.

Given the heterogeneity of interventions, designs, and outcomes of the studies that fit the inclusion criteria, we used a narrative synthesis methodology to analyze results. Studies included in the analysis were assessed for quality using checklists compatible with the study design and methodology: the Cochrane Collaboration Manual for randomized control trials,^16^ the STROBE statement for observational studies,^17^ and The Joanna Briggs Institute’s Checklist for Qualitative Research.^18^ Eligible articles were given a quality score that was converted into the percentage of the total achievable score. As per previous reports, thresholds were set up to rate studies’ quality as low (0%–33%), medium (34%–66%), or high (67% or above).^10^ Articles were divided between NL, LB, LC, SL, and LV, independently rated for quality, then reviewed in pairs. After peer discussion, if a consensus could not be reached, then a third reviewer rated the article.

## RESULTS

**FIGURE 2:**
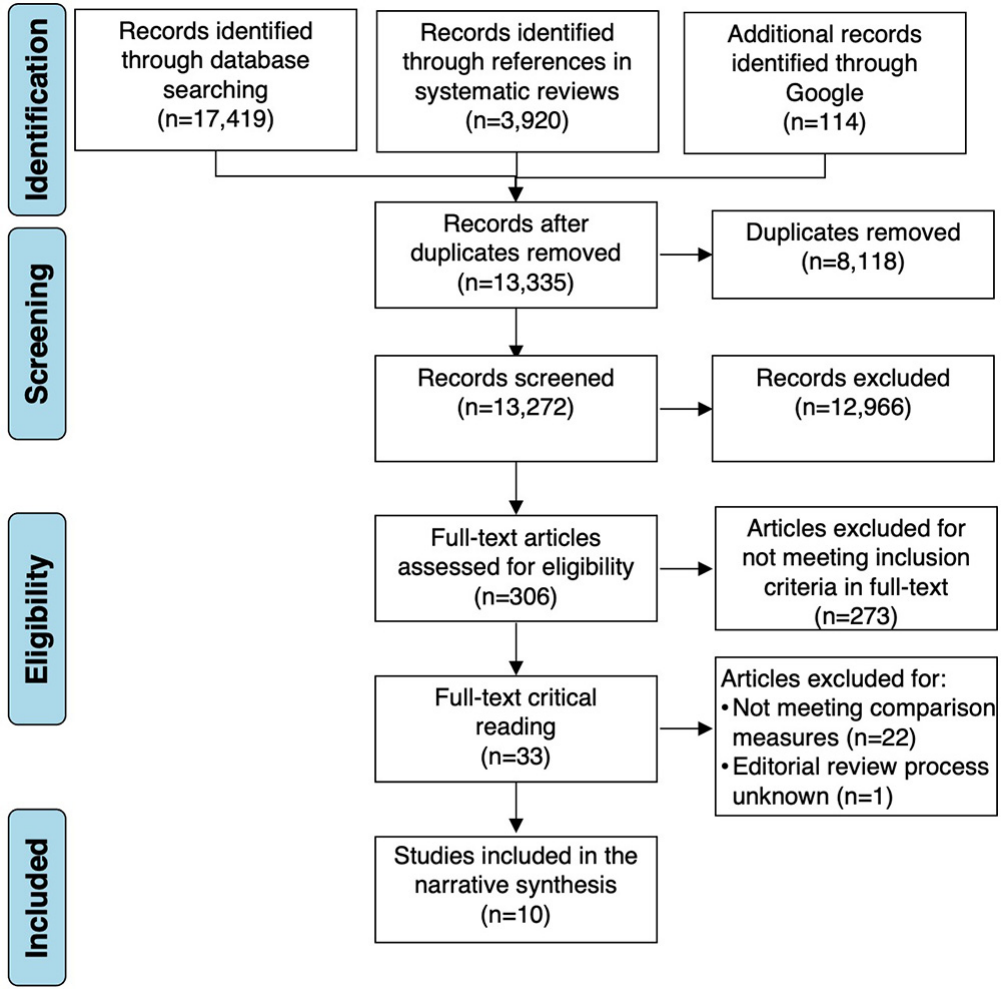
Selection Process for Systematic Review on Interventions to Improve the Reproductive Health of Undocumented Female Migrants and Refugees in Protracted Situations

We obtained a total of 21,453 citations, of which 17,419 corresponded to titles and abstracts obtained through specialized search engines, 114 through the intentional search in Google, and 3,920 through screening the references of relevant systematic reviews (Figure 2). Of the total citations, 8,118 were excluded due to double counting and 12,966 were excluded in the abstract screening for not meeting the inclusion criteria. In addition, using the search word “transit” yielded a large number of unfavorable results related to transport or digestive processes in medical sciences. We assessed 306 articles for eligibility for full-text reading, of which 273 were excluded for not meeting the population, intervention, and outcome criteria. Thirty-three articles were included for a full-text critical reading, 23 of which did not meet the inclusion criteria because they lacked a measure of comparison or impact estimate (n=22) or because the data were found on an NGO website, and it was not known whether the data went through an editorial review process (n=1).^19^ Additional reasons for exclusion were gaps in data analysis and other methodological weaknesses, such as small sample size (<30 participants) or insufficient data regarding the intervention. Ten studies were included in the narrative synthesis (Table 2).^20^^–^^29^

**TABLE 2.** Characteristics, Interventions, and Results of Studies To Improve Reproductive Health for Undocumented Female Migrants and Refugees

**Study and Assessed Quality**	**Study Characteristics**	**Study Intention**	**Outcomes Related to Reproductive Health**	**Intervention Impact Results** ^a^
Fuentes-Afflick^b^ (2006)^20^	Design: Cross-sectional survey	To compare effect of state of residence and immigration status on the use of PNC among Hispanic women in CA, NY, and FL compared to U.S. born citizens in NY after 1996 PRWORA was imposed, which prohibited using federal Medicaid funds for undocumented immigrants for publicly funded services. CA and NY continued to provide services with nonfederal resources; FL fully adopted PRWORA measures	Use of prenatal care services Adequate: initiated during the first trimester and 6 ANC visits or more Inadequate: initiated after first trimester and fewer than 6 ANC visits	75% of UMW in CA and NY received prenatal care in the first trimester vs. 57% of UMW in FL 35% of UMW in FL had fewer than 6 prenatal visits vs. CA (13%) and NY (12%) UMW in CA and FL entered ANC approximately 2 weeks later than those in NY UMWs had higher likelihood of inadequate use of ANC than U.S.-born citizens in CA, NY, FL but odds ratio was greater in FL (3.5) compared to CA (1.9) and NY (1.6)
	Population: Postpartum Hispanic women (aged 17 years and older) (64% undocumented)			
	Location: USA (CA, NY, and FL)			
Raheel^b^ (2012)^21^	Design: Cross-sectional survey	To measure effect of health subsidy on uptake of contraception among refugees, who were assigned to 2 NGOs: One NGO provided 90% subsidies for health care (subsidized care) One NGO only encouraged refugees to use public and private health care resources (nonsubsidized care)	Knowledge, attitudes, and practices regarding FP Approval of FP by women, friends, and spouses	Has ever heard of FP (nonsubsidized vs. subsidized): 44.9% vs. 88.9% Currently using any contraceptive method (nonsubsidized vs. subsidized): 24.9% vs. 54.5% Approval of FP (nonsubsidized vs. subsidized): Women: 49.5% vs. 85.8% Friends: 47.7% vs. 85.5% Spouses: 44.6% vs. 88.6%
	Population: Currently married Afghan female refugees (aged 15–49 years) and their families			
	Location: Karachi, Pakistan			
Purdin^c^ (2008)^22^	Design: Secondary analysis	To reduce maternal mortality among Afghan refugees in the Hangu district of Pakistan Established EmOCs staffed with female Pakistani doctors, nurse-midwives, and dayas from among the refugee population. EmOC staff are available or on-call 24/7 Educated on danger signs during pregnancy and the importance of skilled attendance Facilitation of referral forms to EmOC	Decreased maternal mortality ratio and maternal deaths during birth Increased utilization of services (EmOC): births, complications, referrals, cesarean deliveries Case fatality rate Coverage Knowledge of danger signs among the target population	Maternal mortality ratio per 100,000 (before vs. after): 291 vs. 102 Percentage of refugee births in an EmOC facility (1996 vs. 2007): 4.8% vs. 67.2% Cesarean delivery fatality among refugees: 0.2% vs. United Nations target of less than 1% Prenatal coverage (3+ PNC visits) from 2000 to 2006: 49% vs. 90%
	Population: Afghan female refugees (N=96,300 distributed in 11 refugee camps)			
	Location: Refugee camps in Hangu district, Pakistan			
Truppa^b^ (2019)^23^	Design: Mixed-method, 2 cross-sectional post-intervention surveys (HS vs. CS)	To determine if program was enabling access to essential primary health care services for the most vulnerable populations residing in catchment areas heavily affected by the Syrian crisis and determined through various indicators obtained through HS or CS	Use of FP, ANC, and delivery care services	HS: (Lebanese women vs. Syrian refugees) Percentage of women who sought but did not obtain contraception (54.11% vs. 43.6%) Percentage of women that attended ANC visits (63.18% vs. 70.26%) Median number of ANC visits (7 vs. 4) CS (Lebanese vs. Syrian refugees): Percentage of women that attended ANC visits (87.5% vs. 98.6%) Median of ANC visits (4 vs. 4)
	Population: Lebanese and Syrian refugee women (aged 18–50 years) and caretakers of minors (both genders)			
	Location: Lebanon			
Tousaw^b^ (2017)^24^	Design: Qualitative interviews post-intervention (n=22); secondary data analysis of SARP records (N=81)	To document experiences of women who accessed SARP: Trained counselors in Thailand and Burma in pregnancy options counseling, skill-building exercises, and logistics of SARP SARP offered referrals for care and financial coverage	Successful referrals Denial of services to referred women Experiences and satisfaction of women with the SARP	52/81 successful referrals (64%) 17/81 referrals that were denied service (21%) Satisfactory experience with SARP services: Comprehensive RH services Women described SARP counselors as understanding, open, friendly and nonjudgmental Women reported a sense of empowerment and commitment to advocating for SARP
	Population: Migrant and refugee women from Burmese communities			
	Location: Northern Thailand			
Tousaw^b^ (2018)^25^	Design: In-depth qualitative interviews (n=16) Secondary analysis of program records (N=918)	To provide early MA in low-resource and legally restricted setting Trained social workers and counselors (network providers) on counseling and provision of early MA Provided access to a free supply of misoprostol for early MA Made network providers available for questions, concerns, and follow-up within 4 weeks of initial dose	Percentage of successful complete MA Experiences of self-managed abortions	96% of women had successful complete MA Qualitative results: Positive experiences regarding safety, tolerable side effects, and the absence of complications Satisfaction with the counseling services provided Women’s concerns about the legal risks for themselves and the network providers for involvement in MA
	Population: Female refugees and migrants from Burma with self-reported pregnancy of 9 weeks or less			
	Location: Thailand-Burma border			
McGinn^d^ (2006)^26^	Design: Cross-sectional post-intervention survey with RHG-exposed and nonexposed refugees	To increase literacy skills, knowledge of RH, and use of RHS available in the camps Literacy sessions with reproductive health content: safe motherhood, FP, STI, HIV/AIDS, and GBV Literacy sessions were conducted by previously trained teachers. Sessions had a medium duration of 2 hours, twice a week for a duration of between 6 months and 5 months	Increased knowledge and use of contraception, as well as discussion about contraception use with partners Increased knowledge of STIs Antenatal care, including immunization Knowledge of PNC	Difference between before vs. after of having ever spoken to their partners or family members about (before vs. after)^e^: RH: 18% (69% vs. 87%) Condoms: 20% (65% vs. 85%) STIs: 31% (54% vs. 85%) HIV: 18% (66% vs. 84%)
	Population: Female refugees from Liberia and Sierra Leone, either illiterate or with up to 2 years of formal education (N=2,325)			
	Location: Guinea			
Rosenberg^d^ (2017)^27^	Design: Qualitative study with focus groups	To train refugees engaged in sex work to become peer educators to meet the RH needs of refugees performing sex work Trained 2 cohorts of peer educators on human rights, sex work, SRH, life skills, community outreach and advocacy Peer educators designed and implemented community outreach activities (distributing condoms, capacity-building sessions, peer counseling and support sessions)	Self-reported increased knowledge on SRH, legal topics related to sex work and GBV Increased willingness to share the knowledge with their peers	Peer educators reported an increase in knowledge (e.g., safe sex and FP) Peer educators reported perceiving themselves as ambassadors for female refugees engaged in sex work
	Population: Female refugees engaged in sex work (N=50 peer educators)			
	Location: Kampala, Uganda			
Howard^b^ (2011)^28^	Design: Cross-sectional post-intervention survey	To improve RH of refugee women, refugee providers created RHG, which was integrated into local health system Trained health care facilitators to conduct outreach/education activities with women, men, and youth^a^ and provide information and advice on RH, and distribute condoms and spermicides^a^ Created drama groups and youth-oriented activities to engage larger audiences and spread RH messages^a^	Attitudes on PNC Knowledge regarding: Reason to attend PNC Danger signs in pregnancy Actions if danger signs present Utilization of services by parous women	Nonsignificant differences reported in outcomes between women in intervention group vs. those who were not
	Population: Female refugees (aged 15–49 years) from Sierra Leone and Liberia living in 48 refugee camps			
	Location: Guinea Forest Region			
Stevens^b^ (2018)^29^	Design: Mixed-method study with surveys and focus groups	To increase FA uptake and decrease NTD incidence among migrant and refugee women Workshops with health workers on NTDs and dosing of FA Community outreach through posters, pamphlets, and workshops with different stakeholders (e.g., local HCWs, men, NGOs, and field managers)	Uptake of FA among migrant and refugee women Increased knowledge about FA among HCWs	Negative difference in FA uptake before and after the intervention (1.3% vs. 0.65%; *P*=.465) Percentage of HCWs that knew that NTDs could be prevented by taking FA before conception (16% before vs. 72% after; *P*<.001)
	Population: Pregnant female migrant and refugees seeking care (N=371) and HCWs (N=100)			
	Location: Thailand-Myanmar border			

Abbreviations: ANC, antenatal care; CA, California; CHW, community health workers; CS, clinic survey; EMOC, emergency obstetric care center; FA, folic acid; FHW, female health workers; FL, Florida; FP, family planning; GBV, gender-based violence; HS, household survey; HCW, health care worker; MA, medical abortion; NGO, nongovernmental organization; NTD, neural tube defects; NY, New York; PNC, postnatal care; PRWORA, Personal Responsibility and Work Opportunity Reconciliation Act; RH, reproductive health; RHG, reproductive health group; SARP, Safe Abortion Referral Program; SRH, sexual and reproductive health; STI, sexually transmitted infection; UMW, undocumented migrant women.

^a^ Information obtained from an additional article cited in Howard et al.^28^

^b^ Study rated high quality according to measures used.

^c^ Study rated low quality according to measures used.

^d^ Study rated medium quality according to measures used.

^e^ It is not clear in the study results if women at baseline and follow-up were the same women.

All studies included were observational; there were no randomized or field trials. Six studies were implemented in refugee camps,^21^^–^^23^^,^^26^^–^^28^ and the other 4 studies included undocumented migrant women and refugees in destination countries^20^ and border areas.^24^^,^^25^^,^^29^ There were 5 quantitative,^20^^–^^22^^,^^26^^,^^28^ 3 qualitative,^24^^,^^25^^,^^27^ and 2 mixed-methods studies.^23^^,^^29^ As shown in Table 2, 7 studies were rated high quality according to the measures used.^20^^,^^21^^,^^23^^–^^25^^,^^28^^,^^29^ However, in most cases, the methodological design was not adequate to report impact results, as they were ex post facto studies and/or did not have a comparison group. The 10 studies were conducted in the United States,^20^ Pakistan,^21^^,^^22^ Lebanon,^23^ Thailand,^24^^,^^25^^,^^29^ Guinea,^26^^,^^28^ and Uganda.^27^

Nine studies were conducted by teams working in refugee camps or by university-led research groups. The interventions were diverse: 2 evaluated the improvement in access to RH services through the provision of public (Medicaid)^20^ or private health insurance^21^; 2 studies evaluated the impact of improving health infrastructure and staff capacities in border areas^22^^,^^23^; 2 evaluated referral services and/or access to safe abortion^24^^,^^25^; and 4 evaluated the impact of educational interventions, implemented either by trained personnel or peers, on increasing RH knowledge (Table 2).^26^^–^^29^

Two financial support intervention studies done in the United States^20^ and Pakistan^21^ showed that enabling undocumented migrant and refugee women to obtain RH services for free or at low cost promotes RH service utilization. The study in Pakistan showed that, compared to only making health services available, subsidizing health care through public or private insurance plans doubled the frequency of a woman having heard about contraceptive methods (44.9% vs. 88.9%), and increased approval of contraceptive use among women, their friends, and partners.^21^ Likewise, the use of contraceptive methods increased by 29 percentage points in the group of women with access to subsidized health care, compared to the group of women who did not have access to health care subsidies.^21^ Similarly, the U.S. study showed that, compared to migrant women in Florida state where Medicaid was restricted for this population, the proportion of pregnant undocumented migrant women that attended prenatal care during the first trimester was 18 percentage points higher in the states of California and New York, where subsidized services through Medicaid were provided.^20^ In addition, among migrant women with access to Medicaid, 22% more women received adequate prenatal services compared to those without access to Medicaid.^20^

Two financial support intervention studies done in the United States and Pakistan showed that enabling undocumented migrant and refugee women to obtain RH services for free or at low cost promotes RH service utilization.

Two studies conducted in Pakistan^22^ and Lebanon^23^ showed that health systems interventions that established or strengthened health service delivery structures targeting refugees or located in refugee areas generally had a positive impact on reproductive and maternal health. In Pakistan, the establishment of emergency obstetric care units combined with community-level efforts to educate refugee and undocumented migrant women and their families and facilitate links with the unit was related to a 41% increase in the number of women that attended at least 3 prenatal visits, a reduction in maternal mortality (291 vs. 102 per 100,000 women), and a 0.2% prevalence in maternal deaths after cesarean delivery.^22^ The prevalence of maternal deaths related to cesarean deliveries was consistent and within the <1% goal of the United Nations at that time.^22^ In Lebanon, strengthening primary health care services in areas with a high population of refugees showed high RH service utilization among refugees; in some health areas, service use was higher among refugee women than local women in the same area (Table 2).^23^ This intervention is described as “supporting” facilities, but no further detail was provided.^23^ These 2 interventions occurred during a period of at least 3 years and included aspects of cultural and gender sensitivity.^22^^,^^23^

The 2 studies conducted in Thailand focused on facilitating access to safe abortion for migrant women.^24^^,^^25^ One evaluated a referral program to access free safe abortion services.^24^ The other evaluated an intervention with community health promoters, who provided counseling on safe abortion and access to a free supply of oral misoprostol.^25^ Both interventions showed positive results in terms of referral to services (64% of all women who requested the referral were given one)^24^ and effectiveness of the intervention (96% of women who accessed misoprostol had a complete abortion).^25^ Women expressed satisfaction with the initiative and a perception of increased empowerment and capacity to advocate for their RH rights after the intervention. One study informed that the intervention with oral misoprostol was safe and the side effects reported were tolerable.^25^ The 2017 study described that one-fifth of participants reported that they were denied abortion services mainly due to the subjective interpretation of health personnel on legal grounds.^24^ Both interventions had an important subsidy component; the costs of medical supplies, transportation, and interpreters were absorbed by the implementing organizations and not by the women themselves.

Four studies conducted in Guinea,^26^^,^^28^ Uganda,^27^ and Thailand^29^ evaluated the influence of educational interventions on RH outcomes of migrant and refugee women. All but 1 of the studies included a qualitative component in the evaluation.^28^ Two educational interventions trained community promoters and implemented community outreach strategies.^28^^,^^29^ One included an educational campaign that raised awareness among health care workers and women (including refugees) on the prevention of neural tube defects with the intake of folic acid during the early stages of pregnancy.^29^ The other study trained refugee women to provide RH education, referrals, and contraceptives for women in their communities.^28^ The third followed a peer-education methodology to increase the knowledge of migrant women sex workers regarding human rights, life skills, advocacy, sex work, and sexual and RH.^27^ The last study evaluated the impact of RH literacy sessions on refugee women living in camps.^26^

These studies suggested variable effects of the interventions on knowledge and behaviors, with higher gains in the former. While 1 study showed a significant increase in knowledge among health care workers on the importance of folic acid intake, it did not observe a corresponding uptake in folic acid consumption by women.^29^ Another study identified a mean difference of 21.7% in the socialization with partners and other family members of knowledge acquired by women regarding safe sex.^26^ In contrast, there were no statistically significant differences in antenatal care seeking between women who participated in interventions and those who did not^28^ nor in increased folic acid consumption practices.^29^ The 4 study findings suggested that educational interventions can increase knowledge, especially when led by peers, but knowledge shifts do not always translate into changes in practices or increased care-seeking behaviors. Still, promoting safe spaces to learn about or discuss RH seems to provide a sense of self-confidence,^26^ to inspire women to become advocates of their RH,^27^ and is particularly relevant for stigmatized populations.^27^ Among migrant sex workers and transgender women, peer education helped address concerns of privacy and confidentiality, reporting increased access to information, management skills, and ability to discuss and disseminate knowledge with peers and clients alike.^27^

## DISCUSSION

Our review of literature from the past 2 decades identified 10 observational studies that, according to our inclusion criteria, provided sufficient documentation and measures of intervention impacts on RH of migrant women and female refugees. Interventions can be broadly classified as: (1) interventions that subsidized or gave financial assistance for accessing RH services at zero or minimum cost^20^^,^^21^; (2) health systems interventions that established or strengthened service structures for refugees in the areas where they live or facilitated access to specific RH services, such as abortion information and counseling and postabortion care;^22^^–^^25^ and (3) educational and community interventions that aim to increase RH knowledge and thus help-seeking behaviors and access to RH services.^26^^–^^29^

Promising approaches for future research and programming interventions in protracted situations have a focus on geographic proximity, are cost-sensitive, involve opportunities for peer sharing and learning, and are culturally appropriate. Additionally, interventions must recognize and address undocumented migrant concerns around legality and access to resources.

One reason for the limited number of studies evaluating intervention impacts on RH outcomes in refugee women is that most interventions were implemented in adverse political environments and contexts that may pose unique challenges for study purposes.

For example, in our study, of the 6 countries with reported studies, none favor broad and free access to comprehensive RH services for refugees and undocumented migrants. Restrictions are present in access to pregnancy termination and a wide range of contraceptives.^30^^–^^34^ Furthermore, these countries show a trend toward the privatization of health services, a punitive culture toward the free exercise of sexuality, and the criminalization of sex work.^35^^–^^40^ In some instances, the countries that temporarily host migrants or refugees are neighboring states to those in conflict and are susceptible to forced migration implications (e.g., Ghana, Lebanon, and Pakistan). These unstable circumstances directly affect the implementation of interventions and the collection of data required for research and evaluation.^26^^,^^28^ Nonetheless, some studies indicate the possibility of documenting the impact of intervention through quantitative and qualitative study designs. Future implementation research should consider both what is pragmatic and flexible in protracted migration settings.

Findings from this systematic review suggest that providing free or low-cost services to migrant women and female refugees in protracted situations is a critical strategy in successfully ensuring access to the continuum of sexual and reproductive, maternal, and newborn care services. The findings also concur with other studies documenting increased use of contraceptive methods and prenatal care following interventions.^6^ Studies with nonmigrant populations similarly report that increased enrollment in economic transfer programs or a reduced cost of services results in increased utilization of reproductive and prenatal services.^41^^,^^42^ Subsidies reduce financial barriers to accessing health services and necessary medical supplies,^43^^,^^44^ as well as potentially motivate migrants who may otherwise hesitate to spend out-of-pocket given the precarious socioeconomic circumstances that they are in. Also, when the subsidy is public, undocumented migrants are provided with conditions similar to those of citizens, facilitating migrant women's ability to exercise their right to RH care.^45^

Findings from this review suggest that providing free or low-cost services to migrant women and female refugees in protracted situations is a critical strategy in successfully ensuring access to the continuum of sexual and reproductive, maternal, and newborn care services.

Review findings coincide with other studies around the need to strengthen health systems structures to ensure access to available health services among refugee and undocumented migrant women. Staffing health care units with culturally competent and trained personnel influences the prevalence of contraception use and reduces maternal and neonatal complications and adverse outcomes.^11^^,^^12^^,^^46^^,^^47^ Studies included in our review do not adequately describe aspects important for the replication of interventions. For example, the establishment of a unit of care entails hiring qualified, trained, and certified personnel; thus, studies should document financial considerations and mechanisms for sustainability. A study in Tanzania with a nonmigrant population identified a median per-patient cost of US$290 across 6 evaluated facilities. The 2 items associated with the highest expenditure were the hiring of personnel and the purchase of equipment (32% and 28% of the total budget, respectively).^48^^,^^49^ A mention of the minimum budget required to implement and maintain these interventions in border areas and/or with refugee and migrant populations would be helpful to those aiming at replicating specified intervention dimensions. Even though the budget to be allocated in each country would be different, a good estimation exercise would consist of observing the proportion of the budget allocated to RH services compared to the total expenditure on health in the countries that have already shown favorable impacts on RH outcomes among undocumented female migrants and female refugees in protracted situations.

Our review of access to comprehensive RH services, such as abortion information and counseling and postabortion care, aligns with findings of studies with nonmigrant populations regarding the safety and effectiveness of involving trained lay health care workers as facilitators of access,^50^ particularly for migrant women who face similar legal RH restrictions as reported by nonmigrant women but have additional concerns such as deportation and needs such as understanding and navigating the host country’s health system and overcoming language barriers and cultural differences regarding service provision.^24^ In addition, the relevance of establishing a referral system to enable health care facility access to comprehensive RH services is implicit. Tousaw et al. is an example of the feasibility of establishing referral systems for accessing services that are not always readily accessible to migrant women.^24^ Future studies on interventions facilitating refugees' and undocumented migrants' access to comprehensive RH services through lay workers, however, should discuss practical, ethical, and legal specificities while training personnel, as any attempt to replicate these initiatives will benefit from such considerations.

Although we acknowledge that education-focused interventions are important to empower women to act on the knowledge they have gained, our review points to educational interventions as potentially having a greater impact if implemented after or concurrently with health system capacity improvements (e.g., infrastructure and specialized staff). According to the results of this systematic review, interventions aimed at increasing RH knowledge raise awareness but do not necessarily influence behavioral change.^26^^,^^27^^,^^29^ Interventions that link and conduct follow-up with service users whenever health care capacities are already installed show better results in RH outcomes of migrant and refugee women^51^ and of nonmigrant women alike.^52^ Moreover, the inclusion of nearby communities and women’s partners in educational interventions can be beneficial for women, as these allies become facilitators (and not barriers) to adopting health care–seeking behaviors.^53^ Lastly, educational interventions providing information show a self-reported empowerment effect on participants, especially when the intervention includes the creation of safe spaces and peer-support groups.^26^^,^^27^ Thus, educational interventions, combined with community-level strategies and availability of health services, can increase the probability of RH self-care or self-efficacy in the short, medium, and long term.^54^

One common element that emerged across study narratives was that cultural competency and security should inform intervention design and implementation. We affirm this need and recommend that these elements are included as essential aspects of future intervention design and evaluations. Examples from the interventions analyzed include naming the emergency obstetric care unit a “minor operating theater” to respect refugee women’s desire for privacy^22^ or reconsidering the use of the label “sex worker,” being that it is often a temporary and forced source of income that women do not use to define themselves.^27^

One common element that emerged across study narratives was that cultural competency and security should inform intervention design and implementation.

As reviewed studies included primarily monitoring and auditing data, there is a need for investigators to support as much as possible a health system culture of de-identified electronic record keeping, provided that the security and confidentiality of the data can be guaranteed—particularly in conflict areas, as records can get lost during violent attacks.^22^^,^^26^^,^^28^^,^^55^ Our review also shows that local social conflict may arise when interventions are not inclusive of all of the population living in a locality, such as some refugees receiving subsidized care while others do not^21^ or refugees receiving more care than uninsured local populations.^23^ The latter is particularly more visible among refugees (compared to undocumented migrants), given the financial support that countries might receive from the UN Refugee Agency.^55^

Our review’s identification of prevailing gaps suggests that further evaluation is needed to inform the states and the community of practice aiming to improve RH outcomes of migrant and refugee women living in protracted situations. Particularly important is evidence on how to address difficulties that arise because of the mobility of this population and the constant changes in staff providing health care in these contexts.

In addition, although our review shows the pivotal role that NGOs have in providing access to RH for migrants and refugees in protracted situations, the responsibility of states to provide comprehensive RH services to all women remains. Evaluating interventions and publishing results can help in enticing states to get involved in the initiatives and fulfill their responsibilities. For instance, 2 of the 10 included studies commented on the creation of collaborations between NGOs and public health care institutions or government officials, after the interventions' results became available.^21^^,^^22^ How data from evaluating interventions implemented by NGOs influence public policy and government actions in these contexts is an area that merits further exploration.

Other areas to explore include considering telehealth and digital platforms for information on RH topics, service options, and self-care to improve RH outcomes and other innovative interventions, as well as implementation science and participatory approaches. Studies included in this review do not mention the referral systems they use to provide comprehensive care, nor do they mention a trauma-informed approach to the RH services offered. Referral to mental health services is particularly important in the migratory context since migration is often related to traumatic violence experiences before or during the migratory journey. Similarly, future implementation and evaluation of RH interventions should consider a trauma-informed approach for RH (i.e., providing a trustworthy environment and ensuring care does not revictimize service users). Such approaches can be part of the training on cultural sensitivity for health care providers and other personnel working with undocumented and refugee populations. Trauma-informed approaches and resulting outcomes should be assessed using implementation research.

Documenting, assessing, and evaluating efforts to improve undocumented migrant women and female refugees’ RH outcomes—possibly drawing on perspectives of these populations—are needed to strengthen care provision. A knowledge management platform for quick referencing could further allow NGOs and governmental institutions working toward improving RH outcomes in migrant and refugee populations to explore what other interventions have been implemented, where they have been implemented, and the results of these interventions.

Documenting, assessing, and evaluating efforts to improve undocumented migrant women and female refugees’ RH outcomes are needed to strengthen care provision.

### Limitations

Our review has several limitations. First, this was a secondary study that directly depended on the contents and quality of data from the primary studies. As already mentioned, many studies did not include in-depth evaluations of the impact of interventions on the RH outcomes of migrant and refugee women. Quasi-experimental and pre-post evaluations with a control population group could shed more light on the impact of interventions on the target population. However, given the challenges of conducting rigorous evaluations among these populations, elevating the voices of successful experiences and conducting pragmatic implementation research should be prioritized in partnership with the community of practice. Obtaining information on the differential outcomes that interventions may achieve on subpopulations (e.g., adolescent versus adult women) could also provide indications on how to improve practice. Another limitation was that because the focus of the inclusion criteria was on RH, interventions aimed at addressing other SRH needs, such as sexual violence and sexually transmitted infections, were beyond the study’s scope, even though these needs are highly prevalent among migrant communities.^56^ Further studies should focus on the impact of interventions to address gender-based violence and sexually transmitted infections among this target population. Additionally, the search strategies were done in 3 languages, and it is possible that we could have found more studies if the number of languages had been expanded. Finally, the heterogeneity of the data did not allow for reaching summary measures through a meta-analysis. Nevertheless, this heterogeneity revealed some of the diverse populations, contexts, and circumstances of mobile communities worldwide. Our findings represent a good portion of the variety of implementation contexts for NGOs and governmental institutions, allowing our conclusions and recommendations to be transferrable.

## CONCLUSION

During the migratory process, undocumented migrant women and female refugees in protracted situations find themselves in contexts that put them at higher risk of sexual violence and unwanted RH outcomes. There is limited high-quality research on migrant-centered RH interventions. However, interventions that reduce disease, unintended pregnancy, and fatal RH outcomes include subsidizing or giving financial assistance for accessing RH services and establishing or strengthening health services, including referral services, for undocumented migrant women and female refugees where they live. The successes and challenges to providing RH services identified within this review can inform future programming worldwide, particularly in regions like Latin America and the Caribbean, where fewer interventions have been implemented and evaluated. Intervention budgets and RH service financing should include evaluation as an essential element of future programmatic research. This review, consistent with other studies, suggests combining interventions in a culturally acceptable comprehensive approach will best meet the RH needs of migrant and refugee women.

## Notes

**Peer Reviewed**

## References

[1] International Organization for Migration (IOM). *World Migration Report 2022*. IOM; 2022. Accessed November 16, 2022. https://publications.iom.int/books/world-migration-report-2022

[2] Chavez Baray SM, Moya E, Ravelo Blancas P, Báez Ayala SL. Experiencias de violencias de mujeres migrantes en Ciudad Juárez y El Paso. In: *Migración y Salud*. Consejo Nacional de Población; 2020:61–73.

[3] Freedman J, Crankshaw TL, Mutambara VM. Sexual and reproductive health of asylum seeking and refugee women in South Africa: understanding the determinants of vulnerability. Sex Reprod Health Matters. 2020;28(1):1758440. 10.1080/26410397.2020.1758440. 32425112 PMC7888032

[4] Urquia ML, Glazier RH, Gagnon AJ, et al; ROAM Collaboration. Disparities in pre‐eclampsia and eclampsia among immigrant women giving birth in six industrialised countries. BJOG. 2014;121(12):1492–1500. 10.1111/1471-0528.12758. 24758368 PMC4232918

[5] Vik ES, Aasheim V, Schytt E, Small R, Moster D, Nilsen RM. Stillbirth in relation to maternal country of birth and other migration related factors: a population-based study in Norway. BMC Pregnancy Childbirth. 2019;19(1):5. 10.1186/s12884-018-2140-3. 30611227 PMC6321699

[6] Almeida LM, Caldas J, Ayres-de-Campos D, Salcedo-Barrientos D, Dias S. Maternal healthcare in migrants: a systematic review. Matern Child Health J. 2013;17(8):1346–1354. 10.1007/s10995-012-1149-x. 23334357

[7] Hasstedt K, Desai S, Ansari-Thomas Z. *Immigrant Women’s Access to Sexual and Reproductive Health Coverage and Care in the United States.* Guttmacher Institute; 2018. Accessed December 1, 2022. https://www.guttmacher.org/article/2018/11/immigrant-womens-access-sexual-and-reproductive-health-coverage-and-care-united 30458586

[8] Cheng IH, Advocat J, Vasi S, et al. A Rapid Review of Evidence-Based Information, Best Practices and Lessons Learned in Addressing the Health Needs of Refugees and Migrants: Report to the World Health Organization. 2018. Accessed December 1, 2022. https://cdn.who.int/media/docs/default-source/documents/publications/a-rapid-review-of-evidence-based-information-health-of-refugees-and-migrantsf2d00add-c78a-4fd6-9b51-bf7c033b81d4.pdf

[9] Warren E, Post N, Hossain M, Blanchet K, Roberts B. Systematic review of the evidence on the effectiveness of sexual and reproductive health interventions in humanitarian crises. BMJ Open. 2015;5(12):e008226. 10.1136/bmjopen-2015-008226. 26685020 PMC4691726

[10] Jennings L, George AS, Jacobs T, Blanchet K, Singh NS. A forgotten group during humanitarian crises: a systematic review of sexual and reproductive health interventions for young people including adolescents in humanitarian settings. Confl Health. 2019;13(1):57. 10.1186/s13031-019-0240-y. 31788022 PMC6880589

[11] Singh NS, Aryasinghe S, Smith J, Khosla R, Say L, Blanchet K. A long way to go: a systematic review to assess the utilisation of sexual and reproductive health services during humanitarian crises. BMJ Glob Health. 2018;3(2):e000682. 10.1136/bmjgh-2017-000682. 29736272 PMC5935157

[12] Singh NS, Smith J, Aryasinghe S, Khosla R, Say L, Blanchet K. Evaluating the effectiveness of sexual and reproductive health services during humanitarian crises: a systematic review. PLoS One. 2018;13(7):e0199300. 10.1371/journal.pone.0199300. 29980147 PMC6035047

[13] International Organization for Migration (IOM). *Glossary on Migration*. IOM; 2019. Accessed December 1, 2022. https://publications.iom.int/system/files/pdf/iml_34_glossary.pdf

[14] Starrs AM, Ezeh AC, Barker G, et al. Accelerate progress—sexual and reproductive health and rights for all: report of the Guttmacher– Lancet Commission. Lancet. 2018;391(10140):2642–2692. 10.1016/S0140-6736(18)30293-9. 29753597

[15] Moreno MA, Molina N. La intervención social como objeto de estudio: discursos, prácticas, problematizaciones y propuestas. Athenea Digit. 2018;18(3):e2055. 10.5565/rev/athenea.2055

[16] Higgins JPT, Green S, eds. *Cochrane Handbook for Systematic Reviews of Interventions*. Version 5.1.0 [updated March 2011]. The Cochrane Collaboration; 2011. Accessed December 1, 2022. https://es.cochrane.org/sites/es.cochrane.org/files/uploads/Manual_Cochrane_510_reduit.pdf

[17] STROBE. Strengthening the reporting of observational studies in epidemiology. Accessed October 24, 2022. https://www.strobe-statement.org/

[18] The Joanna Briggs Institute (JBI). *Checklist for Qualitative Research*. JBI; 2017. Accessed December 1, 2022. https://jbi.global/sites/default/files/2019-05/JBI_Critical_Appraisal-Checklist_for_Qualitative_Research2017_0.pdf

[19] Reproductive health. Mae Tao Clinic. Updated August 21, 2019. Accessed December 1, 2022. https://maetaoclinic.org/our-services/health-services/reproductive-health/

[20] Fuentes-Afflick E, Hessol NA, Bauer T, et al. Use of prenatal care by Hispanic women after welfare reform. Obstet Gynecol. 2006;107(1):151–160. 10.1097/01.AOG.0000191299.24469.1b. 16394053

[21] Raheel H, Karim MS, Saleem S, Bharwani S. Knowledge, attitudes and practices of contraception among Afghan refugee women in Pakistan: a cross-sectional study. PLoS One. 2012;7(11):e48760. 10.1371/journal.pone.0048760. 23133658 PMC3487847

[22] Purdin S, Khan T, Saucier R. Reducing maternal mortality among Afghan refugees in Pakistan. Int J Gynaecol Obstet. 2009;105(1):82–85. 10.1016/j.ijgo.2008.12.021. 19232603

[23] Truppa C, Leresche E, Fuller AF, et al. Utilization of primary health care services among Syrian refugee and Lebanese women targeted by the ICRC program in Lebanon: a cross-sectional study. Confl Health. 2019;13(1):7. 10.1186/s13031-019-0190-4. 30923560 PMC6420751

[24] Tousaw E, La RK, Arnott G, Chinthakanan O, Foster AM. “Without this program, women can lose their lives”: migrant women’s experiences with the Safe Abortion Referral Programme in Chiang Mai, Thailand. Reprod Health Matters. 2017;25(51):58–68. 10.1080/09688080.2017.1392220. 29210341

[25] Tousaw E, Moo SNHG, Arnott G, Foster AM. “It is just like having a period with back pain”: exploring women’s experiences with community-based distribution of misoprostol for early abortion on the Thailand–Burma border. Contraception. 2018;97(2):122–129. 10.1016/j.contraception.2017.06.015. 28780239

[26] McGinn T, Allen K. Improving refugees’ reproductive health through literacy in Guinea. Glob Public Health. 2006;1(3):229–248. 10.1080/17441690600680002. 19153909

[27] Rosenberg JS, Bakomeza D. Let’s talk about sex work in humanitarian settings: piloting a rights-based approach to working with refugee women selling sex in Kampala. Reprod Health Matters. 2017;25(51):95–102. 10.1080/09688080.2017.1405674. 29231800

[28] Howard N, Woodward A, Souare Y, et al. Reproductive health for refugees by refugees in Guinea III: maternal health. Confl Health. 2011;5(1):5. 10.1186/1752-1505-5-5. 21486433 PMC3080804

[29] Stevens A, Gilder ME, Moo P, et al. Folate supplementation to prevent birth abnormalities: evaluating a community-based participatory action plan for refugees and migrant workers on the Thailand-Myanmar border. Public Health. 2018;161:83–89. 10.1016/j.puhe.2018.04.009. 29935473 PMC6086336

[30] Maruf F, Tappis H, Lu E, Yaqubi GS, Stekelenburg J, Akker T van den. Health facility capacity to provide postabortion care in Afghanistan: a cross-sectional study. Reprod Health. Published online 2019:1–24. 10.21203/rs.2.18903/v1 34321023 PMC8317397

[31] Fathallah Z. Moral work and the construction of abortion networks: women’s access to safe abortion in Lebanon. Health Hum Rights J. 2019;21(2):21–31. Accessed December 1, 2022. https://www.hhrjournal.org/2019/12/moral-work-and-the-construction-of-abortion-networks-womens-access-to-safe-abortion-in-lebanon/PMC6927363 31885433

[32] NARAL Pro-Choice America Foundation. *Who Decides? The Status of Women’s Reproductive Rights in the United States*. 21st ed. NARAL; 2012. Accessed December 1, 2022. https://www.prochoiceamerica.org/wp-content/uploads/2017/04/2012-Who-Decides.pdf

[33] Center for Reproductive Rights. *Women’s Reproductive Rights in the United States: A Shadow Report*. Center for Reproductive Rights; 2006. Accessed December 1, 2022. https://reproductiverights.org/wp-content/uploads/2020/12/US-HR-Comittee-2006.pdf

[34] Chaturachinda K, Boonthai N. Unsafe abortion: an inequity in health care, Thailand perspective. J Popul Soc Stud. 2017;25(3):287–297. 10.25133/JPSSv25n3.007

[35] Camara M, Camara A, Camara N. The healthcare system in Africa: the case of Guinea. Int J Community Med Public Health. 2015;2(4):685–689. 10.18203/2394-6040.ijcmph20150933

[36] Ramesh M, Wu X. Realigning public and private health care in southeast Asia. Pac Rev. 2008;21(2):171–187. 10.1080/09512740801990238

[37] Kronfol NM. Rebuilding of the Lebanese health care system: health sector reforms. East Mediterr Health J. 2006;12(3–4):459–473. 17037717

[38] El-Jardali F, Fadlallah R, Matar L. *Primary Health Care Systems (PRIMASYS): Comprehensive Case Study from Lebanon*. WHO; 2017. Accessed December 1, 2022. https://apps.who.int/iris/handle/10665/341171

[39] Woolhandler S, Himmelstein DU, Ahmed S, et al. Public policy and health in the Trump era. Lancet. 2021;397(10275):705–753. 10.1016/S0140-6736(20)32545-9. 33581802

[40] Tumwesige Ateenyi F, du Toit L, Mwebaza E, Zalwango F, Nanyange J. *Legal Regulation of Sex Work in Uganda: Exploring the Current Trends and Their Impact on the Human Rights of Sex Workers*. Jjuuko A, ed. Human Rights Awareness and Promotion Forum; 2016.

[41] Ross R, Fagan T, Dutta A. *Is Health Insurance Coverage Associated with Improved Family Planning Access? A Review of Household Survey Data from Seven FP2020 Countries*. Palladium, Health Policy Plus; 2018. Accessed December 1, 2022. http://www.healthpolicyplus.com/ns/pubs/10253-10458_FPUHCReview.pdf

[42] Were LPO, Were E, Wamai R, Hogan J, Galarraga O. Effects of social health insurance on access and utilization of obstetric health services: results from HIV+ pregnant women in Kenya. BMC Public Health. 2020;20(1):87. 10.1186/s12889-020-8186-y. 31959153 PMC6971983

[43] Comfort AB, Peterson LA, Hatt LE. Effect of health insurance on the use and provision of maternal health services and maternal and neonatal health outcomes: a systematic review. J Health Popul Nutr. 2013;31(4)(Suppl 2):81–105. 10.3329/jhpn.v31i4.2361. 24992805

[44] Wang W, Temsah G, Mallick L. The impact of health insurance on maternal health care utilization: evidence from Ghana, Indonesia and Rwanda. Health Policy Plan. 2017;32(3):366–375. 10.1093/heapol/czw135. 28365754 PMC5400062

[45] Chiarenza A, Dauvrin M, Chiesa V, Baatout S, Verrept H. Supporting access to healthcare for refugees and migrants in European countries under particular migratory pressure. BMC Health Serv Res. 2019;19(1):513. 10.1186/s12913-019-4353-1. 31337406 PMC6651950

[46] Ameh CA, Mdegela M, White S, van den Broek N. The effectiveness of training in emergency obstetric care: a systematic literature review. Health Policy Plan. 2019;34(4):257–270. 10.1093/heapol/czz028. 31056670 PMC6661541

[47] Gabrysch S, Nesbitt RC, Schoeps A, et al. Does facility birth reduce maternal and perinatal mortality in Brong Ahafo, Ghana? A secondary analysis using data on 119 244 pregnancies from two cluster-randomised controlled trials. Lancet Glob Health. 2019;7(8):e1074–e1087. 10.1016/S2214-109X(19)30165-2. 31303295 PMC6639244

[48] Mengistu T, Berruti A, Krivelyova A, Swor M, Waite R, Maro G. Cost of providing emergency obstetric care in Tanzania’s Kigoma region. Int J Health Plann Manage. 2019;34(4):e1510–e1519. 10.1002/hpm.2820. 31270861 PMC6904496

[49] Banke-Thomas A, Wilson-Jones M, Madaj B, van den Broek N. Economic evaluation of emergency obstetric care training: a systematic review. BMC Pregnancy Childbirth. 2017;17(1):403. 10.1186/s12884-017-1586-z. 29202731 PMC5716021

[50] World Health Organization (WHO). *Health Worker Roles in Providing Safe Abortion Care and Post-Abortion Contraception*. WHO; 2015. Accessed December 1, 2022. https://apps.who.int/iris/handle/10665/181041 26401543

[51] Chamberlain C, O’Mara-Eves A, Porter J, et al. Psychosocial interventions for supporting women to stop smoking in pregnancy. Cochrane Database Syst Rev. 2017;2020(3):CD001055. 10.1002/14651858.CD001055.pub5. 28196405 PMC6472671

[52] Banerjee SK, Andersen KL, Warvadekar J, Pearson E. Effectiveness of a behavior change communication intervention to improve knowledge and perceptions about abortion in Bihar and Jharkhand, India. Int Perspect Sex Reprod Health. 2013;39(03):142–152. 10.1363/3914213. 24135046

[53] Blanc AK. The effect of power in sexual relationships on sexual and reproductive health: an examination of the evidence. Stud Fam Plann. 2001;32(3):189–213. 10.1111/j.1728-4465.2001.00189.x. 11677692

[54] Lassi ZS, Bhutta ZA. Community-based intervention packages for reducing maternal and neonatal morbidity and mortality and improving neonatal outcomes. Cochrane Database Syst Rev. 2015;2015(3):CD007754. 10.1002/14651858.CD007754.pub3. 25803792 PMC8498021

[55] Von Roenne A, Von Roenne F, Kollie S, Swaray Y, Sondorp E, Borchert M. Reproductive health services for refugees by refugees: an example from Guinea. Disasters. 2010;34(1):16–29. 10.1111/j.1467-7717.2009.01112.x. 19459901

[56] Araujo JDO, Souza FM, Proença R, Bastos ML, Trajman A, Faerstein E. Prevalence of sexual violence among refugees: a systematic review. Rev Saude Publica. 2019;53:78. 10.11606/s1518-8787.2019053001081. 31553381 PMC6752644

